# Obesity and occupation in Thailand: using a Bayesian hierarchical model to obtain prevalence estimates from the National Health Examination Survey

**DOI:** 10.1186/s12889-021-10944-0

**Published:** 2021-05-13

**Authors:** Jongjit Rittirong, John Bryant, Wichai Aekplakorn, Aree Prohmmo, Malee Sunpuwan

**Affiliations:** 1 Institute for Population and Social Research, Mahidol University, Salaya, Phutthamonthon, Nakhorn Pathom, 73170 Thailand; 2 Bayesian Demography Limited, 9 Buscot Gate, Christchurch, 8042 New Zealand; 3 Faculty of Medicine, Ramathibodi Hospital, Mahidol University, Bangkok, Thailand

**Keywords:** Obesity, Occupation, Thailand, Bayesian hierarchical model, Small area estimation

## Abstract

**Background:**

Like many developing countries, Thailand has experienced a rapid rise in obesity, accompanied by a rapid change in occupational structure. It is plausible that these two trends are related, with movement into sedentary occupations leading to increases in obesity. National health examination survey data contains information on obesity and socioeconomic conditions that can help untangle the relationship, but analysis is challenging because of small sample sizes.

**Methods:**

This paper explores the relationship between occupation and obesity using data on 10,127 respondents aged 20–59 from the 2009 National Health Examination Survey. Obesity is measured using waist circumference. Modelling is carried out using an approach known as Multiple Regression with Post-Stratification (MRP). We use Bayesian hierarchical models to construct prevalence estimates disaggregated by age, sex, education, urban-rural residence, region, and occupation, and use census population weights to aggregate up. The Bayesian hierarchical model is designed to protect against overfitting and false discovery, which is particularly important in an exploratory study such as this one.

**Results:**

There is no clear relationship between the overall sedentary nature of occupations and obesity. Instead, obesity appears to vary occupation by occupation. For instance, women in professional occupations, and men who are agricultural or fishery workers, have relatively low rates of obesity.

**Conclusion:**

Bayesian hierarchical models plus post-stratification offers new possibilities for using surveys to learn about complex health issues.

## Background

Thailand has, in recent decades, experienced a rapid rise in obesity, with 42% of women and 33% of men having a BMI greater than 25 kg/m^2^ in 2014 [1]. This rise has coincided with a movement into sedentary occupations. Between 1990 and 2011, the proportion of workers employed in agriculture shrank from 63 to 41%, while the proportion employed in the services sector rose from 23 to 40% [2].

Work is an important component of overall physical activity, even if it is not the only one. A study in Barbados [3], for instance, found the work make up 57% of total physical activity energy expenditures. Evidence from high-income countries suggests that more sedentary occupations with less physical activity may be associated with higher obesity, though the relationship between occupation and obesity is confounded with socio-economic status especially education, and can differ between women and men [4–15].

Estimating how obesity rates vary across the Thai population is useful for understanding the recent changes, and for designing a policy response. Evidence on the relationship between occupation and obesity is, however, limited, in Thailand, and in middle-income countries more generally.

Constructing detailed estimates of Thai obesity rates is challenging. Thailand undertakes a National Health Examination Survey (NHES) every 5–10 years, which includes measures of obesity [16–19]. But once the sample has been disaggregated by occupation and by geographical and demographic variables, the number of observations in each cell is small, and direct estimates of prevalence are unreliable.

In this paper, we use Bayesian hierarchical models [20] to estimate obesity prevalence from data from the 2009 National Health Examination Survey. Our most detailed estimates are disaggregated by occupation, age, sex, education, region within Thailand, and urban-rural residence, though disaggregation to the level is driven partly by the need to account for the complex design of the Health Examination Survey. In our analyses, we work with population-weighted averages of the most detailed estimates. Findings from the analysis are of substantive interest, given that, internationally, there is limited information about the demographic and occupational profile of obesity within middle-income countries. The paper also illustrates how modern statistical methods can be used to obtain detailed prevalence estimates from household surveys. Our analysis is exploratory. We seek to understand the relationship between obesity and occupation, how this relationship varies with age, sex, and education level, and whether sedentary occupations have higher obesity than non-sedentary ones.

## Methods

### Data

Our main data source is the 2009 round of the Thai National Health Examination Survey (NHES). The survey uses a complex design, with stratification and clustering based on region and village or urban community. Interviews were conducted by local health personnel. The dataset we use in our analysis consists of records for 10,127 working-age (20–59 years) adults. Table 1 shows the unweighted sample classified by age, education, urban-rural residence, occupation, and obesity.

**Table 1:** Respondents in the 2009 National Health Examination Survey

	Female (n)	Male (n)	Female (%)	Male (%)
Age				
20–29	781	764	14.3	16.4
30–39	1369	1144	25.0	24.6
40–49	1807	1431	33.0	30.8
50–59	1523	1308	27.8	28.1
Education				
Primary or lower	3287	2437	60.0	52.4
Secondary or higher	2179	2202	39.8	47.4
Missing	14	8	0.3	0.2
Urban-rural residence				
Urban	3129	2462	57.1	53.0
Rural	2347	2182	42.8	47.0
Missing	4	3	0.1	0.1
Occupation				
Sedentary				
Legislators, senior officials, and managers	3	12	0.1	0.3
Professionals	207	145	3.8	3.1
Technicians and associate professionals	1273	1071	23.2	23.0
Clerical support workers	214	110	3.9	2.4
Non-Sedentary				
Service workers and shop and market sale workers	215	145	3.9	3.1
Elementary occupations	1182	1188	21.6	25.6
Skilled agricultural and fishery workers	1053	1155	19.2	24.9
Armed forces	1	89	0.0	1.9
Plant and machine operators and assemblers	31	163	0.6	3.5
Unknown				
No occupation	1064	330	19.4	7.1
Other	214	228	3.9	4.9
Missing	23	11	0.4	0.2
Obesity (Asian cutoff)				
Non-Obese	2898	3646	52.9	78.5
Obese	2575	995	47.0	21.4
Missing	7	6	0.1	0.1
Obesity (European cutoff)				
Non-Obese	4269	4429	77.9	95.3
Obese	1204	212	22.0	4.6
Missing	7	6	0.1	0.1
Total	5480	4647	100.0	100.0

The classification of occupations in Table 1 is based on categories from the International Labour Organization classification ISCO-88 [21]. The survey asked the respondents’ current occupation, without distinguishing between permanent and temporary occupations. In our analyses, we divide occupations into two groups – sedentary and non-sedentary – based on the classification suggested by Choi et al. [22] as shown in Table 2. Choi et al. derive their classification from an analysis of detailed data from the Korean Working Conditions Survey. We assume that, particularly in industries other than agriculture, work conditions in Korea are broadly similar to those in Thailand.

**Table 2:** Classification of occupations as sedentary and non-sedentary

Sedentary	Non-Sedentary	Unknown
Legislators, senior officials, and managers	Service workers and shop and market sale workers	No occupation
Professionals	Elementary occupations	Other
Technicians and associate professionals	Skilled agricultural and fishery workers	
Clerical support workers	Armed forces	
	Plant and machine operators and assemblers	

We measure obesity using waist circumference. Waist circumference is generally a more sensitive measure of risks of cardiovascular disease and premature mortality than the Body Mass Index (BMI), in that it is less affected by individual variation in lean muscle mass [1, 16, 23–25]. In the NHES, the trained local health personnel measured each respondent’s waist circumference by placing a flexible plastic tape under the lower rib and 2 cm over the navel while respondents breathed normally. Two measurements were taken, and the average of the two was recorded. As with the BMI, nutritionists recommend using different thresholds to define obesity in different populations. International Diabetes Federation (IDF) [26] and Alberti et al. [27] recommend using a threshold of 90 cm for Asian men, 80 cm for Asian women, 102 cm for European men, and 88 cm for European women. We adopt the Asian standard, but as a sensitivity test we repeat our analyses using the European standard.

As can be seen in Table 1, the NHES dataset contains a small number of missing values. We impute these values using ‘k-nearest neighbour imputation’ as implemented in the function kNN in R package VIM [28]. This method searches the dataset for records that most closely resemble the ones with missing values, and borrows values from those.

We use the official One Percent Sample from Thailand’s 2010 Population and Housing Census to calculate population weights. The One Percent Sample is constructed by the National Statistical Office, using stratified random sampling. As with our obesity data, we calculate population weights for a classification defined by age, sex, education, urban-rural residence, region, and occupation.

### Statistical models

We estimate obesity prevalence using a Bayesian hierarchical model [20]. The model allows us to obtain sensible estimates, including uncertainty measures, even for combinations of variables with few observations. It does this by ‘pooling strength’ across the entire dataset, and by smoothing estimates based on sample size.

Our model in effect assumes that, within each combination of variables, people in our sample are a simple random sample of people in the target population. To satisfy this assumption, our model includes all the variables that affect people’s probability of being selected into the sample, even when these variables are not of substantive interest in our study. However, rather than use these very detailed results, we use population weights derived from the population census to aggregate up to a classification containing only the variables of interest. Results for this more aggregate classification have less uncertainty than the original highly-disaggregated results. The general technique of fitting a very detailed hierarchical model to account for the complex survey design, and then using population weights to aggregate up is known as Multilevel Regression and Post-Stratification (MRP) [29, 30].

The first layer of our model states that the number of people with obesity in each cell within our classification by age, sex, education, occupation, urban-rural residence, and region can be treated as a draw from a binomial distribution,

$$ {y}_i\sim Binomial\left({n}_i,{\pi}_i\right). $$

Subscript *i* indexes cell within the classification: for instance, *i* might refer to 20–29 year old females with secondary or more education, with a “technicians and associated professional” occupation, living in an urban area in northern Thailand. The levels of all classifying variables are shown in Table 1. The quantity *n*_*i*_ is the number of respondents in cell *i*; *y*_*i*_ is the number of respondents in cell *i* who are obese; and *π*_*i*_ is the probability that a randomly-chosen person in cell *i* is obese. We use *π*_*i*_ as our measure of prevalence, and we seek to estimate it for all *i*. We include education in the model because we expect at least some of any bivariate relationship between occupation and obesity to reflect the influence of education, rather than of occupation itself.

Altogether, our classification includes 1760 cells. Once obesity counts *y*_*i*_ are disaggregated to this degree, 56% of cells have value 0. The median count among non-zero cells is 2 and the mean is 4.7. The median count of respondents across all *n*_*i*_ is 1 and the mean is 5.8. With such small numbers, modelling individual-level randomness in obesity outcomes, as we do with the binomial distribution, is essential.

The NHES is stratified by region and by urban-rural residence, implying that a person’s probability of being included in the survey varies according to the person’s region, and whether the person lives in an urban or rural area. By including region and urban-rural residence in our model, we allow for these differences in selection probabilities.

The NHES is also clustered, meaning that, to save collection costs, the survey selects communities at random within each stratum, and samples intensively within each community. We do not explicitly model the clustering process, as it would complicate the analysis considerably. If outcomes are correlated within communities even after controlling for background characteristics, then, by omitting these correlations, our model is understating uncertainty. However, because our model allows for variation by age, sex, occupation and education, and all combinations of these variables, it should do a good job of controlling for background characteristics, so the understatement of uncertainty due to clustering should be relatively small.

In the second layer of the model, we apply a logit transform to *π*_*i*_ and treat the transformed value as a draw from a normal distribution.

$$ logit\left({\pi}_i\right)\sim N\left({x}_i\beta, {\sigma}^2\right) $$

Vector *β* includes main effects for age, sex, education, occupation, urban-rural residence, and region. It also includes all second-order interactions between age, sex, education, and occupation. The presence of the second-order interactions implies, for instance, that the age-profile for obesity is expected to be different for male and females, and that the occupational profile is expected to be different for people with and without secondary education. The vector *x*_*i*_ is the *i* th row of the design matrix. It consists entirely of 1 s and 0 s, and is constructed so that each cell *i* receives the appropriate combination of main effects and interactions.

In the third layer of the model, each main effect or interaction is treated as a draw from a normal distribution with mean 0 and standard deviation *τ*. For instance, the main effect for age is modelled using

$$ {\beta}_a^{age}\sim N\left(0,{\tau}_{age}^2\right). $$

Probabilistic sub-models like this are known within Bayesian statistics as prior distributions. Prior distributions can serve various purposes, but here they are acting as a soft constraint on the size of the main effects and interactions. The prior distributions pull the final estimates for the main effects and interactions towards zero. When an estimate is based on many observations, this pull is relatively unimportant, but when an estimate is based on only a few observations, the use of a prior distribution can have a strong moderating influence on the final estimate. This moderating influence, which is sometimes referred to as regularization or shrinkage, provides protection against over-fitting and false discovery [31].

The strength of the pull towards zero is governed by the standard deviation term *τ*. When a main effect or interaction has three levels or fewer (as with the sex effect, for instance), so that there is little scope for estimating *τ*, we set it equal to 1. When a main effect or interaction has four or more levels, we treat it as a draw from a half-normal distribution, that is, a normal distribution restricted to non-negative values,

$$ {\tau}_{age}\sim {N}^{+}\left(0,{A}_{age}^2\right). $$

The scale term *A* in the half-normal distributions is set equal to 1 for main effects and to 0.5 for interactions. Lower values for *A* tend to produce lower values for *τ* and hence generate a stronger pull towards 0 and more regularisation. Our choice of values for *A* is based on the assumption that main effects tend to be larger than interactions [32]. The standard deviation term *σ* is assumed to be drawn from a half-normal distribution with scale 1.

The outcome variable for our main model is obesity based on the Asian standard for waist circumference. However, as a sensitivity test, we also fit a model using obesity based on the European standard. We fit both models using function estimateModel in R package demest, available at github.com/statisticsnz/demest. All code for the analysis is available at [link to github repository.] The function estimateModel uses a set of techniques known as Markov chain Monte Carlo (MCMC) to generate its estimates [20].

The output from the modelling is a set of draws from the “posterior distribution” for the model parameters. If *θ* denotes a particular parameter in our model, then we obtain a sample *θ*^(1)^, *θ*^(2)^, …, *θ*^(*S*)^ of draws for *θ*. To obtain a point estimate for *θ* we use the median of value for the *θ*^(*s*)^; to obtain a 95% credible interval (the Bayesian analogue of a confidence interval), we use the 2.5 and 97.5% quantiles for *θ*^(*s*)^.

To calculate a point estimate and credible interval for some function *f* of *θ*, we calculate *f*(*θ*^(1)^), *f*(*θ*^(2)^), …, *f*(*θ*^(*S*)^), and take the median and upper and lower quantiles of these values. The same principle extends to functions of multiple parameters. When calculating point estimates and credible intervals for population-weighted averages, for instance, we calculate weighted averages across each draw of the *π*_*i*_, and then calculate the medians and upper and lower quantiles of the weighted averages.

## Results

**Fig. 1:**
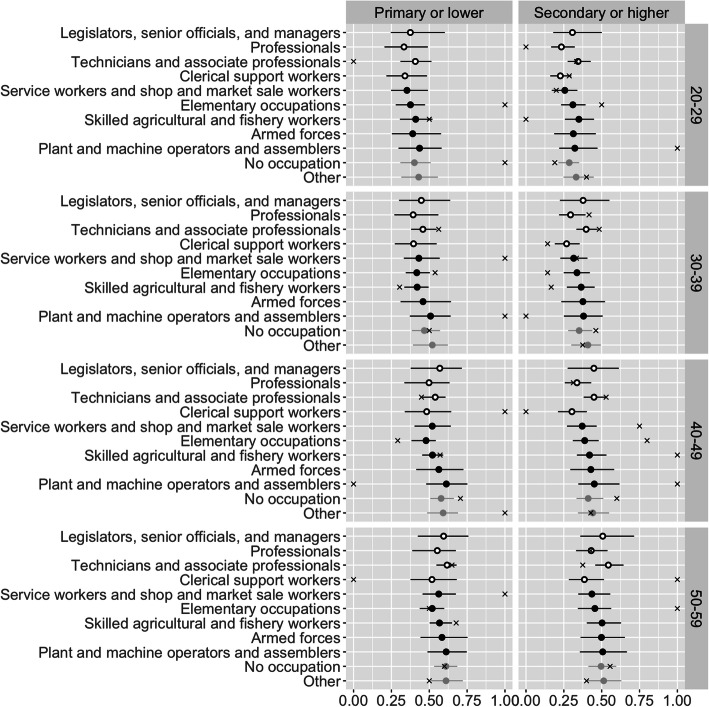
Point estimates and 95% credible intervals for obesity prevalence, for females in urban areas in the Northeast region of Thailand, disaggregated by age, education, and occupation. Open black points denote sedentary occupations, and closed black points denote non-sedentary occupations. Lines denote 95% credible intervals. ‘x’ marks denote direct estimates

The sample from the posterior distribution includes values for all 1760 prevalences *π*_*i*_, plus the parameters from the higher levels of the model. To illustrate, Fig. 1 shows point estimates and 95% credible intervals for *π*_*i*_ for females in urban areas in the Northeast region of Thailand. The ‘x’ marks are direct estimates–that is, the estimates derived by dividing the observed number of obese respondents by the number of respondents, independently for each combination of the classifying variables.

Some patterns are discernible in the point estimates: for instance, obesity seems to rise with age. The widths of the credible intervals vary from occupation to occupation. For example, the credible intervals for “legislators, senior officials, and managers”, for whom there are relatively few observations, are much wider than the credible intervals for “elementary occupations”. Overall, however, the level of uncertainty at this level of disaggregation is too great to form conclusions about any patterns in estimated prevalences. The model estimates are, however, less variable than the direct estimates, illustrating how Bayesian hierarchical models pull estimates towards central values. Note that we would not expect 95% of direct estimates to fall within the 95% credible intervals, as the credible intervals refer to the true, underlying prevalence, rather than the observed proportions, which, with small sample sizes, contain substantial random noise.

**Fig. 2:**
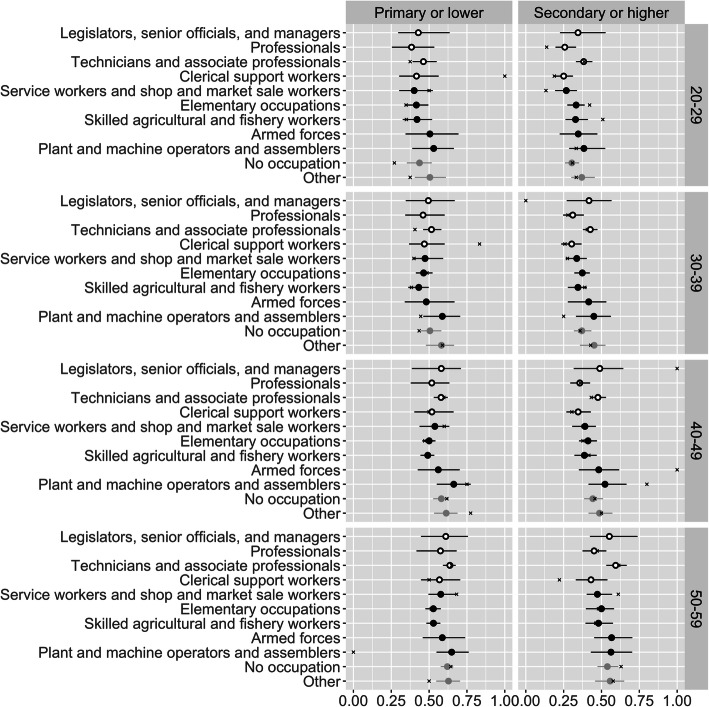
Estimated obesity prevalence by age, sex, education level, and occupation, for females. Open black points denote sedentary occupations, and closed black points denote non-sedentary occupations. Lines denote 95% credible intervals. ‘x’ marks denote direct estimates

Figure 2 shows the result of taking population-weighted averages of the most disaggregated estimates. The figure presents obesity prevalence by age, education level, and occupation, for females. The prevalences average across region and urban-rural residence, and describe patterns at the national level. The figure shows separate results for each occupation, but also distinguishes sedentary occupations from non-sedentary ones.

Most of the credible intervals in Fig. 2 are narrower than those of Fig. 1, since they are based on more data. The exception is credible intervals for combinations of variables that are rare even at the national level, such as women in the “legislators, officials, and managers” category. Obesity appears to rise with age, and to be less common among women with secondary education. However, although there is variation between occupations, there is no clear tendency for sedentary occupations to have higher obesity than non-sedentary group occupations. The results provide some evidence that, among women in sedentary occupations, women in “professional” occupations are less likely to be obese.

The modelled estimates and direct estimates in Fig. 2 are typically close to each other in categories with narrow credible intervals where there is substantial data, and further away in categories with wide credible intervals where there is limited data. When the modelled and direct estimates differ, the modelled ones are more conservative, in the sense that they lie closer to the mean value for each combination of age, sex, and education level. This is an example of the shrinkage or regularization discussed in the Statistical Models section above. In Fig. 2, and more generally, regularisation provides protection against misleadingly extreme estimates based on small samples.

**Fig. 3:**
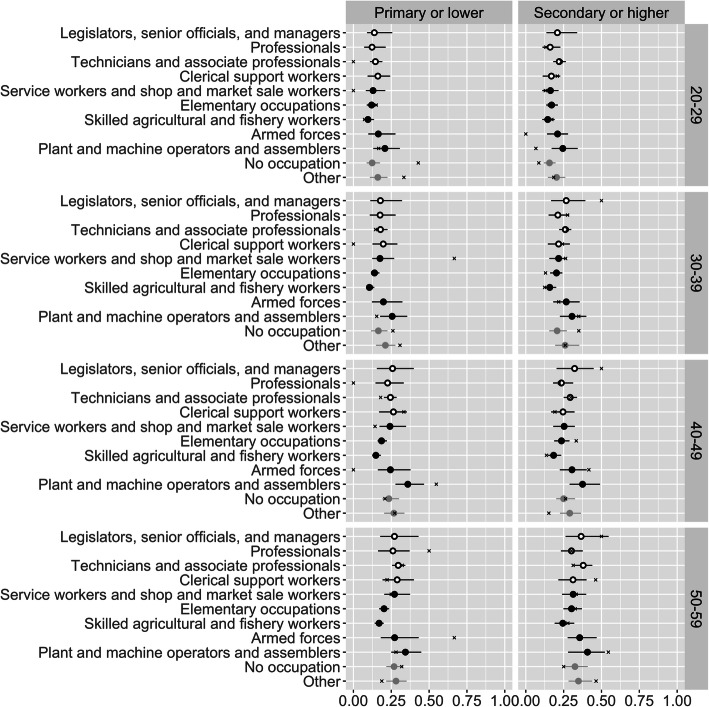
Estimated obesity prevalence by age, sex, education level, and occupation, for males. Open black points denote sedentary occupations, and closed black points denote non-sedentary occupations. Lines denote 95% credible intervals. ‘x’ marks denote direct estimates

Figure 3 is equivalent to Fig. 2, but refers to males rather than females. Obesity is less common among men than among women, though rates among men, like those among women, rise with age. There is once again no clear tendency for sedentary occupations as a whole to have higher obesity rates than non-sedentary occupations. The results do, however, suggest that obesity is relatively low among male “skilled agriculture and fishery workers”.

**Fig. 4:**
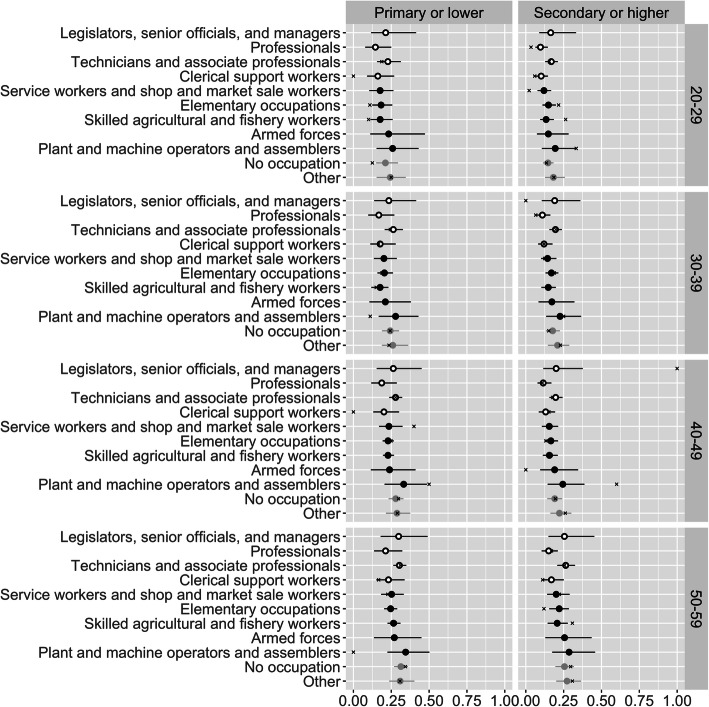
Estimated obesity prevalence using European rather than Asian standards for defining obesity: females. Open black points denote sedentary occupations, and closed black points denote non-sedentary occupations. Lines denote 95% credible intervals. ‘x’ marks denote direct estimates

**Fig. 5:**
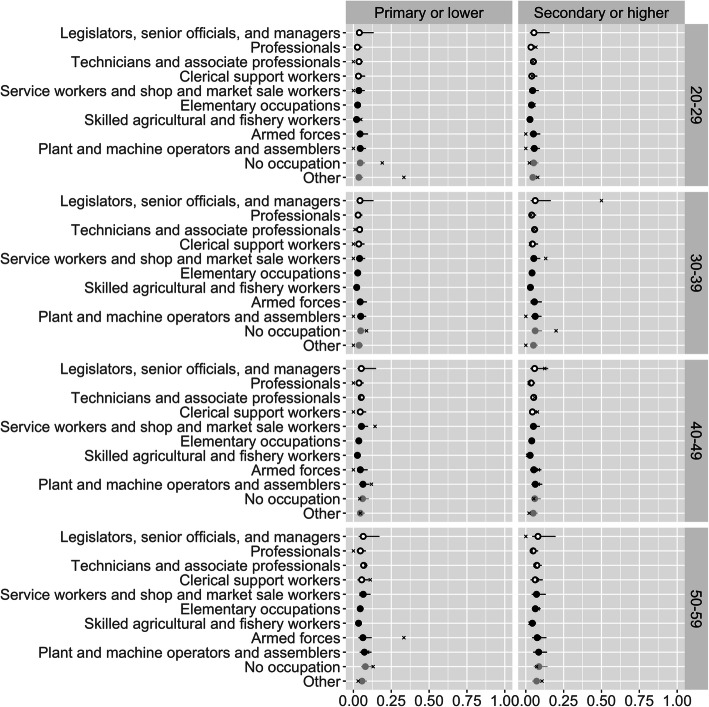
Estimated obesity prevalence using European rather than Asian standards for defining obesity: males. Open black points denote sedentary occupations, and closed black points denote non-sedentary occupations. Lines denote 95% credible intervals. ‘x’ marks denote direct estimates

Using the European standard (Figs. 4 and 5), rather than the Asian standard, to define obesity leads to lower estimated prevalences for both sexes, but particularly for males, as can be seen by comparing Figs. 4 and 5 with Figs. 2 and 3.

Roughly the same patterns appear with the European standard results as appear with the Asian standard results. Among women, there is no overall tendency for sedentary occupations to have higher obesity than non-sedentary occupations, as professional women have relatively low obesity rates. Among men, there is a tendency for agricultural and fishery workers to have low obesity, though the absolute differences in rates are tiny.

## Discussion

Health surveys are a unique source of information about socio-economic differentials in health conditions. When analysing health surveys, however, researchers are constrained by sample size. To unlock the full potential of these surveys, it is necessary to use modern statistical methods that provide sensible estimates even when cell counts are small, such as Bayesian hierarchical models.

Applying Bayesian hierarchical models to 2009 Thai National Health Examination Survey data, in combination with post-stratification based on the 2010 census, we are able to shed light on the complicated relationship between age, sex, education, occupation, and obesity. With traditional statistical approaches, we would need to be concerned that any apparent findings were nothing more than statistical noise. Because of the regularisation build into our model, however, we are protected against such problems.

The results from our analysis suggest that in Thailand there is not a broad relationship, across all occupations, between the sedentary nature of the occupation and the degree of obesity. Instead, obesity varies from occupation to occupation. For instance, among women, professionals have a relatively low obesity rate, despite the relatively sedentary nature of professional work, and even after stratifying on education level. Among men, agricultural and fishery workers are distinctive for their low levels of obesity, even at higher ages.

In future work, we intend to extend our analysis to include more recent rounds of the National Health Examination Survey. This will allow us to see whether the patterns observed for 2009 have persisted. Combining data from multiple surveys will also boost sample sizes, allowing more precise estimates of parameters that are relatively constant over time.

## Conclusions

Bayesian hierarchical models offer new possibilities for using survey data to study complex public health issues. An exploratory analysis of the Thai Health Examination Survey data suggests that there is no simple relationship between the sedentary nature of occupations and the level of obesity. Instead, obesity rates vary across specific occupations, and patterns are different for women and men.

## Data Availability

The data that support the findings of this study are available from Health Systems Research Institute (HSRI) and National Statistical Office (NSO) but restrictions apply to the availability of these data, which were used under license for the current study, and so are not publicly available. Data are however available from the authors upon reasonable request and with permission of HSRI and NSO.

## Abbreviations

BMI: Body Mass Index
HSRI: Health Systems Research Institute
IDF: Institutional Diabetes Federation
MCMC: Markov chain Monte Carlo
MRP: Multiple Regression with Post-Stratification
NHES: National Health Examination Survey
NSO: National Statistical Office

## References

[1] Aekplakorn W, Pakpeankitwatana V, Lee CM, Woodward M, Barzi F, Yamwong S (2007). Abdominal obesity and coronary heart disease in Thai men. Obesity..

[2] National Statistical Office (2017). Working trend of Thai labour.

[3] Howitt C, Brage S, Hambleton IR, Westgate K, Samuels TA, Rose AMC (2016). A cross-sectional study of physical activity and sedentary behaviours in a Caribbean population: combining objective and questionnaire data to guide future interventions. BMC Public Health.

[4] Cameron AJ, Welborn TA, Zimmet PZ, Dunstan DW, Owen N, Salmon J (2003). Overweight and obesity in Australia: the 1999–2000 Australian diabetes, obesity and lifestyle study (Ausdiab). MJA..

[5] Allman-Farinelli MA, Chey T, Merom D, Bauman AE (2010). Occupational risk of overweight and obesity: an analysis of the Australian health survey. J Occup Med Toxicol.

[6] Meseguer CM, Galan I, Herruzo R, Rodríguez-Artalejo F (2011). Trends in leisure time and occupational physical activity in the Madrid region, 1995–2008. Rev Esp Cardiol.

[7] Church TS, Thomas DM, Tudor-Locke C, Katzmarzyk PT, Earnest CP, Rodarte RQ (2011). Trends over 5 decades in U.S. Occupation-related physical activity and their associations with obesity. PLoS ONE.

[8] Monteiro CA, Conde WL, Popkin BM (2002). What has happened in terms of some of the unique elements of shift in diet, activity, obesity, and other measures of morbidity and mortality within different regions of the world?. Public Health Nutr.

[9] Bonauto DK, Lu D, Fan ZJ (2014). Obesity prevalence by occupation in Washington state, behavioral risk factor surveillance system. Prev Chronic Dis.

[10] Caban AJ, Lee DJ, Fleming LE, Gómez-Marín O, LeBlanc W, Pitman T (2005). Obesity in us workers: the national health interview survey, 1986 to 2002. Am J Public Health.

[11] Gans KM, Salkeld J, Risica PM, Lenz E, Burton D, Mello J (2015). Occupation is related to weight and lifestyle factors among employees at worksites involved in a weight gain prevention study. J Occup Environ Med.

[12] Kuntz B, Lampert T (2010). Socioeconomic factors and obesity. Deutsches Arzteblatt international.

[13] Vernay M, Malon A, Oleko A, Salanave B, Roudier C, Szego E (2009). Association of socioeconomic status with overall overweight and central obesity in men and women: the French nutrition and health survey 2006. BMC Public Health.

[14] Wardle J, Waller J, Jarvis MJ (2002). Sex differences in the association of socioeconomic status with obesity. Am J Public Health.

[15] Dėdelė A, Miškinytė A, Andrušaitytė S, Bartkutė Ž (2019). Perceived stress among different occupational groups and the interaction with sedentary behaviour. Int J Environ Res Public Health.

[16] Aekplakorn W, Kosulwat V, Suriyawongpaisal P (2005). Obesity indices and cardiovascular risk factors in Thai adults. Int J Obes.

[17] Aekplakorn W, Hogan MC, Chongsuvivatwong V, Tatsanavivat P, Chariyalertsak S, Boonthum A (2007). Trends in obesity and associations with education and urban or rural residence in Thailand. Obesity..

[18] Aekplakorn W, Inthawong R, Kessomboon P, Sangthong R, Chariyalertsak S, Putwatana P, et al. Prevalence and trends of obesity and association with socioeconomic status in Thai adults: National health examination surveys, 1991–2009. J Obes. 2014. 10.1155/2014/410259. 10.1155/2014/410259 PMC3976913 24757561

[19] Angkurawaranon C, Wisetborisut A, Rerkasem K, Seubsman SA, Sleigh A, Doyle P (2015). Early life urban exposure as a risk factor for developing obesity and impaired fasting glucose in later adulthood: Results from two cohorts in Thailand. BMC Public Health.

[20] Gelman A, Carlin J, Stern H, Dunson DB, Vehtari A, Rubin D (2014). Bayesian data analysis.

[21] International Labour Organization (2016). International Standard Classification of Occupations (ISCO-88). International Standard Classification of Occupations.

[22] Choi SB, Yoon JH, Lee W (2020). The modified international standard classification of occupations defined by the clustering of occupational characteristics in the Korean working conditions survey. Ind Health.

[23] Snijder M, van Dam R, Visser M, Seidell J (2006). What aspects of body fat are particularly hazardous and how do we measure them?. Int J Epidemiol.

[24] Bhurosy T, Jeewon R. Overweight and Obesity Epidemic in Developing Countries: A Problem with Diet, Physical Activity, or Socioeconomic Status? Sci World J. 2014;(2014)964236:7. 10.1155/2014/964236. 10.1155/2014/964236 PMC4212551 25379554

[25] Visscher TL, Seidell JC (2004). Time trends (1993–1997) and seasonal variation in body mass index and waist circumference in the Netherlands. Int J Obes Relat Metab Disord.

[26] (IDF) IDF (2006). The IDF consensus worldwide definition of the metabolic syndrome.

[27] Alberti KG, Zimmet P, Shaw J (2006). Metabolic syndrome–a new world-wide definition. A consensus statement from the international diabetes federation. Diabet Med.

[28] Kowarik A, Templ M (2016). Imputation with the r package vim. J Stat Softw.

[29] Ghitza Y, Gelman A (2013). Deep interactions with MRP: election turnout and voting patterns among small electoral subgroups. Am J Polit Sci.

[30] Downes M, Gurrin LC, English DR, Pirkis J, Currier D, Spittal MJ (2018). Multilevel regression and post stratification: a modeling approach to estimating population quantities from highly selected survey samples. Am J Epidemiol.

[31] Gelman A, Hill J, Yajima M (2012). Why we (usually) don’t have to worry about multiple comparisons. J Res Educ Effective.

[32] Gelman A, Jakulin A, Pittau MG, Su Y-S (2008). A weakly informative default prior distribution for logistic and other regression models. Ann Appl Stat.

